# The role of electrocatalytic materials for developing post-lithium metal||sulfur batteries

**DOI:** 10.1038/s41467-024-49164-6

**Published:** 2024-06-05

**Authors:** Chao Ye, Huan Li, Yujie Chen, Junnan Hao, Jiahao Liu, Jieqiong Shan, Shi-Zhang Qiao

**Affiliations:** 1 School of Chemical Engineering, The University of Adelaide, Adelaide, SA 5005 Australia; 2 Department of Chemistry, City University of Hong Kong, Kowloon, 999077 Hong Kong PR China

**Keywords:** Batteries, Batteries, Batteries

## Abstract

The exploration of post-Lithium (Li) metals, such as Sodium (Na), Potassium (K), Magnesium (Mg), Calcium (Ca), Aluminum (Al), and Zinc (Zn), for electrochemical energy storage has been driven by the limited availability of Li and the higher theoretical specific energies compared to the state-of-the-art Li-ion batteries. Post-Li metal||S batteries have emerged as a promising system for practical applications. Yet, the insufficient understanding of quantitative cell parameters and the mechanisms of sulfur electrocatalytic conversion hinder the advancement of these battery technologies. This perspective offers a comprehensive analysis of electrode parameters, including S mass loading, S content, electrolyte/S ratio, and negative/positive electrode capacity ratio, in establishing the specific energy (Wh kg^−1^) of post-Li metal||S batteries. Additionally, we critically evaluate the progress in investigating electrochemical sulfur conversion via homogeneous and heterogeneous electrocatalytic approaches in both non-aqueous Na/K/Mg/Ca/Al||S and aqueous Zn||S batteries. Lastly, we provide a critical outlook on potential research directions for designing practical post-Li metal||S batteries.

## Abstract (web summary)

Post-lithium metal||S batteries show promise for practical applications, but limited understanding of cell parameters and sulfur electrocatalytic conversion hampers progress. This Perspective provides critical insights on potential research directions for designing practical post-lithium metal||S batteries.

## Introduction

Metal||sulfur (M||S) batteries present significant advantages over conventional electrochemical energy storage devices, including their high theoretical specific energy, cost-effectiveness and the abundant resource of environmentally benign sulfur (S) electrode material^1^. The escalating costs and dwindling resources of lithium have spurred investigations into alternative alkali (earth) and transition metals such as Na, K, Mg, Ca, Zn and Al, as negative electrodes for post-Li M||S batteries^2^. Coupling these materials with S electrodes delivers high theoretical specific energy, such as 1682 Wh kg^−1^ for Mg||S batteries and 1802 Wh kg^−1^ for Ca||S batteries at room temperature^3,4^.

In Na/K||S batteries, the shuttle effect leads to low sulfur-based electrode utilization and inadequate cell Coulombic efficiency (CE). To improve the CE, constructing robust solid electrolyte interphase (SEI) on the metal surface and modifying separators have been reported to suppress the polysulfide shuttling and metal dendrite growth^5^. In ether-based Mg/Ca||S batteries, ionic liquid-based Al||S batteries and aqueous Zn||S batteries, the sluggish dissociation and diffusion kinetics of these multivalent cations result in severe side reactions and passivation issues on the surface of the metal negative electrodes^6^. More importantly, the relatively low solubilities of the sulfur species in these systems further cause the sluggish S conversion kinetics. These kinetical challenges hinder the researchers from achieving post-Li M||S batteries with high CE^3^. Utilizing electrocatalytic materials to reduce the reaction barrier between S and metal cations is a reported method^7–12^. Significant research efforts have explored the use of homogeneous and heterogeneous electrocatalytic materials to improve utilization of S and the CE of the post-Li M||S batteries^13,14^. To demonstrate practical application potential of high-specific-energy post-Li M||S devices, multi-layered pouch cells have been reported, yet the application of electrocatalytic materials for practical M||S battery performance remains inadequately understood^3,15–18^.

Here we establish quantitative parameters including discharge potential, specific capacity and S loading/content in S electrodes, electrolyte dosage and mass of negative electrode materials for boosted device-level specific energy. We also critically appraise advances in applying electrocatalytic materials to boost electrochemical performances of the post-Li M||S batteries and provide perspectives on future research for electrochemical mechanisms under practical environments to inform improved design strategies for electrocatalytic materials, metal negative electrodes and electrolyte solutions in the post-Li M||S battery systems.

### Specific energy evaluation

#### Electrochemical properties of post-Li M||S batteries

**Fig. 1:**
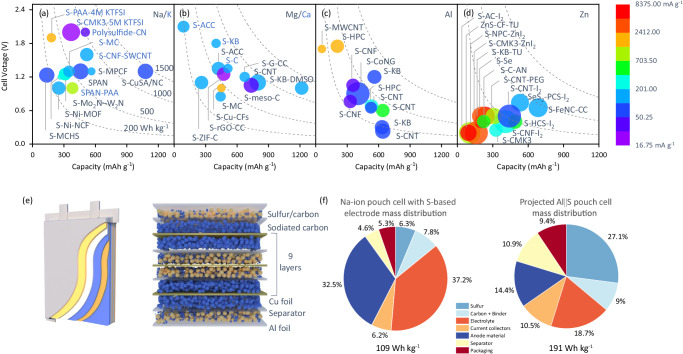
Electrochemical properties for post-Li M||S batteries at electrode level and device level. **a**–**d** Capacity based on sulfur electrode, average discharge cell voltage, rate and S mass loading from 0.2 to 3 mg cm^−1^ in which, larger size refers to greater S loading mass. The acronyms and associated full names are presented in Supplementary Table 1. **e** Na-ion pouch cell with S-based electrodes pouch cell with stacked-cell structure. Reprint from Pampel et al., with permission from Elsevier^26^. **f** The specific energies and mass distributions of the Na-ion pouch cell with S-based electrodes and the Al||S batteries in pouch-cell level. Reprint from Pampel et al., with permission from Elsevier^26^. Pang et al. reproduced with permission from SNCSC^27^.

Electrochemical energy storage properties of electrode materials are evaluated on specified capacity based on capacity of S and the S content in the positive electrode. Reported Na||S batteries generally use nanostructured carbonaceous materials as S hosts, Fig. 1a, demonstrating relatively low electrode capacity of <600 mA h g^−1^ and average discharge cell voltage of 1.0–1.3 V under room temperature and specific current <167.5 mA g^−1^ with S loading of approximately 1 mg cm^−2^ (Supplementary Table 1). Compared with the Na||S system, K||S batteries exhibit a greater average discharge cell voltage of approximately 1.5 V and exhibit lower electrode capacity and corresponding rate. Additionally, the high activity of K causes significant overpotential, leading to a low cycling rate of <168 mA g^−1^.

In comparison with Na||S batteries, Mg||S batteries exhibit comparable electrochemical properties with lower average discharge cell voltage of approximately 1.1 V and rate, C/100 (16.75 mA g^−1^), Fig. 1b. For Ca||S batteries, the theoretical voltage of approximately 2.5 V is amongst the highest of M||S batteries. However, because of practical difficulty in developing compatible electrolytes, studies with limited capacity are reported on Mg||S batteries^19–21^. The Al||S battery, using ionic liquid electrolyte with large ionic size, exhibits low-rate performance. Additionally, the state-of-the-art S content and S loading in laboratory-based Al||S cell studies are approximately 50 m/m% and 1.0 mg cm^−2^, respectively, Fig. 1c^2,4^. Aqueous electrolytes exhibit advantages over non-aqueous electrolytes because of lower cost and higher ionic conductivity at room temperature, making them practical for developing cost-effective and safe battery technologies^22–25^. As shown in Fig. 1d, although the high overpotential of S-to-ZnS conversion leads to average discharge cell voltage of lower than 1.0 V and electrode capacity of aqueous Zn||S batteries, the S loading and the rate performances are highest among the post-Li M||S systems, evidencing the advantage of fast mass transfer in aqueous-based systems. However, the electrode-level specific energy of the aqueous-based system is the lowest among these four systems due to relative low capacity and low average discharge potential.

Although most studies report electrode levels with coin cells, there are those that report M||S batteries at the device level, Fig. 1e. A multi-layer pouch cell using double-side coated S-based electrodes and pre-sodiated hard carbon electrodes exhibited a device-level specific energy of 109 Wh kg^−1^ including the mass of all the device components at room temperature^26^. However, the active material S was 6.3 %, while significant cell mass was represented by an electrolyte (37.2%) and low-capacity negative electrode material (32.5%). The significant portion of negative electrode material was attributed to the use of sodiated hard carbon. Pang *et al*. reported an Al||S battery with NaCl-KCl-AlCl_3_ molten-salt electrolyte operated at moderately elevated temperature (110 °C). The projected Al||S pouch cell configuration includes S content of 27.1 %, resulting in a projected device-level specific energy of 191 Wh kg^−1^^27^. With a high-capacity metal negative electrode, the negative electrode mass ratio was controlled to be 14.4%, as shown in Fig. 1f.

#### Specific energy and quantitative parameters

The specific energy of M||S batteries at device level is dependent on technical parameters including, the S-based positive electrode, S host, electronically conductive additive and binder^28^. Important parameters are therefore, molar mass of S (M_s_), areal mass loading of S (m_sulfur_), mass ratio of S in the positive electrode (R_cathode_), molar mass of the metal negative electrode (M_m_), negative/positive electrode capacity ratio (R_N/P_), areal mass of the electrolyte (m_electrolyte_) and separator (m_separator_, areal mass of 1.0 mg cm^−2^ based on a thickness of 10 μm), areal mass of Al and Cu current collector (m_Al_ and m_Cu_), mass ratio of the package in the ‘whole’ cell (R_package_, 10 m/m%), density of electrolyte (⍴_E_, 1.1 g mL^−1^), and; ratio of electrolyte to S (R_E/S_, μL mg^−1^). The specific capacity of the S electrode can be determined as C_sulfur_. In addition, the mean average discharge cell voltage can be determined as V_mean_, therefore, the device-level specific energy (Wh kg^−1^) is calculated from:

1 $$	{{{{{\rm{Specific\; energy}}}}}}\left({{{{{{\rm{Wh\,kg}}}}}}}^{-1}\right) \\ 	=\frac{{{{{{{\rm{C}}}}}}}_{{{{{{\rm{sulfur}}}}}}}\times {{{{{{\rm{V}}}}}}}_{{{{{{\rm{mean}}}}}}}\times {{{{{{\rm{m}}}}}}}_{{{{{{\rm{sulfur}}}}}}}\times \left(1-{{{{{{\rm{R}}}}}}}_{{{{{{\rm{package}}}}}}}\right)}{\frac{{{{{{{\rm{m}}}}}}}_{{{{{{\rm{sulfur}}}}}}}}{{{{{{{\rm{R}}}}}}}_{{{{{{\rm{cathode}}}}}}}}+\frac{{\,\!}^{{{{{{\rm{m}}}}}}}{{{{{\rm{Al}}}}}}+{\,\!}^{{{{{{\rm{m}}}}}}}{{{{{\rm{Cu}}}}}}}{2}+{{{{{{\rm{m}}}}}}}_{{{{{{\rm{seperator}}}}}}}+{{{{{{\rm{\rho }}}}}}}_{{{{{{\rm{E}}}}}}}\times {{{{{{\rm{R}}}}}}}_{{{{{{\rm{E}}}}}}/{{{{{\rm{S}}}}}}}\times {{{{{{\rm{m}}}}}}}_{{{{{{\rm{sulfur}}}}}}}+\frac{{{{{{{\rm{V}}}}}}}_{{{{{{\rm{s}}}}}}}\times {{{{{{\rm{M}}}}}}}_{{{{{{\rm{m}}}}}}}}{{{{{{{\rm{V}}}}}}}_{{{{{{\rm{m}}}}}}}\times {{{{{{\rm{M}}}}}}}_{{{{{{\rm{s}}}}}}}}\times {{{{{{\rm{R}}}}}}}_{{{{{{\rm{N}}}}}}/{{{{{\rm{P}}}}}}}\times {{{{{{\rm{m}}}}}}}_{{{{{{\rm{sulfur}}}}}}}}$$

**Fig. 2:**
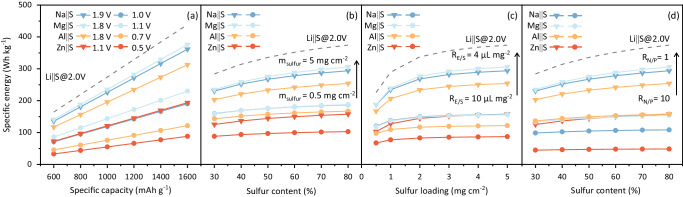
Specific energies and quantitative parameters of the post-Li M||S batteries at device level. **a** Calculated specific energy with capacity and average discharge cell voltage. **b** Calculated specific energy with S content and S loading mass. **c** Calculated S loading mass with the ratio of electrolyte to S (R_E/S_). **d** Calculated specific energy with negative-positive electrode material (R_N/P_) and S content. Dashed lines represent specific energy of Li||S batteries under 2.0 V of average discharge cell voltage with S loading of 5 mg cm^−2^, R_E/S_ of 4 μL mg^−1^ and R_N/P_ of 1.

For a rational comparison of M||S batteries, values are adopted in the calculation including, V_mean_ = 1.9, 1.8, 1.1 and 1.8 V for Na, Mg, Zn, and Al||S battery, respectively, C_sulfur_ = 1300 mAh g^−1^, m_sulfur_ = 5 g cm^−1^, R_cathode_ = 80%, R_N/P_ = 1 and R_E/S_ = 4 μL mg^−1^. The areal mass of Al current collector (m_Al_ = 2.7 mg cm^−2^) is calculated based on a thickness of 10 μm and, half of the current collector mass was used because of the double-sided coating. When metal foil is used as a negative electrode, the current collector of Cu foil can be omitted, resulting in m_Cu_ = 0. The specific capacity, voltage, S loading and S content of selected S electrodes can therefore be established^21,29^. As can be seen in Fig. 2a, increasing specific capacity results in linearly boosted specific energy on the selected M||S batteries. For example, when the specific capacity of Na||S battery increases from 600 to 1200 mAh g^−1^ with an average discharge cell voltage of 1.9 V, the device-level specific energy is boosted from 71 to 190 Wh kg^−1^. The average discharge cell voltage V_mean_ significantly affects device-level specific energy via a linear relationship for all M||S batteries. Increasing S loading m_sulfur_ from 0.5 to 5 mg cm^−2^ results in a significant boost to device-level specific energy from 187 to 294 Wh kg^−1^ for Na||S battery with 80% S content, Fig. 2b.

The impact of R_E/S_ on specific energy can be analyzed against that of S loading in the electrode^26,27^. The impact of S loading on boosting device-level specific energy is limited when it is >2 mg cm^−2^. Therefore R_E/S_ is a major restraining parameter for device-level specific energy of the M||S battery, Fig. 2c. For example, under the S loading of 5 mg cm^−2^, decreasing R_E/S_ from 10 to 4 μL mg^−1^ results in a significant device-level specific energy boost from 157 to 294 Wh kg^−1^ for Na||S battery, in which 4 μL mg^−1^ can be regarded as practical lean electrolyte environment. The impact of R_N/P_ on device-level specific energy can be determined against that of S content, Fig. 2d. For example, if a Na||S battery with a S content 50% and R_N/P_ 10 exhibits a specific energy of 105 Wh kg^−1^, the increasing of S content to 80 % increases specific energy to 109 Wh kg^−1^, whilst decreasing R_N/P_ to 1 result in an increased the specific energy to 294 Wh kg^−1^. This finding evidence that R_N/P_ is an important parameter for high device-level specific energy in M||S batteries. Because of a ‘large’ average discharge cell voltage and high capacity of Li metal, Li||S batteries can exhibit a device-level specific energy of >300 Wh kg^−1^ ^30^. However, the post-Li M||S battery may present a device-level specific energy under 200 Wh kg^−1^ via optimizing S positive electrode, R_N/P_ and R_E/S_^26,31^. The analyses above show that the advantage of the M||S batteries’ high device-level specific energy is conditional. The post-Li M||S batteries might be regarded as alternative solutions for large-scale energy storage instead of a competitor of lithium-based batteries towards ever-rising device-level specific energy.

### Electrocatalytic materials in post-Li M||S batteries

#### Design principles for Electrocatalytic materials

**Fig. 3:**
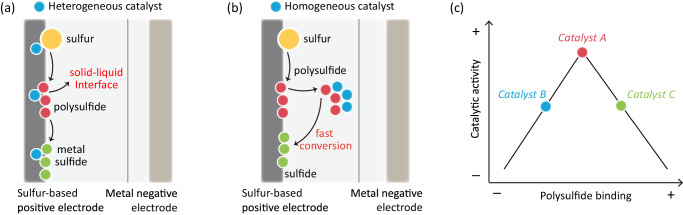
Design for heterogeneous and homogeneous electrocatalytic materials in M||S batteries. Schematic for **a** heterogeneous and **b** homogeneous, catalyst in M||S batteries. (The orange color, red, blue and green spheres represent S, electrocatalyst, polysulfide and metal sulfide, respectively). **c** Volcano plot to demonstrate trade-off between polysulfide binding and catalytic activity of electrocatalysts.

Electrocatalysts for post-Li M||S batteries can generally be categorized into heterogeneous and homogeneous catalysts. Heterogeneous catalysts typically manifest a solid state within S electrode and comprise metals, metal compounds, as well as emerging inorganic and organic complexes. These catalysts are engineered to improve the reversibility of the electrochemical processes at the S electrode|electrolyte interface, thereby aiming to enhance specific capacity, rate and cycling performance, Fig. 3a^32^.

In contrast, homogeneous catalysts are soluble in the electrolyte and enhance redox reactions involving solid phases including formation and oxidation of metal sulfides. By interacting with polysulfides, these catalysts accelerate the kinetics of S conversion and promote effective energy storage and release, Fig. 3b. Figure 3c shows a schematic Volcano plot for rational design of heterogeneous and homogeneous catalysts in the M||S batteries^33^. The weak binding between catalysts and polysulfides, e.g., catalyst B, results in insufficient S conversion and a pronounced shuttle effect. Conversely, the strong chemisorption of polysulfides on catalysts, e.g., catalyst C, might lead to undesired saturation of adsorbed polysulfides and slow conversion. Therefore, a catalyst exhibiting moderate intermediates chemisorption to redox intermediates, coupled with capability to expedite redox reactions, e.g., catalyst A, is preferred. This facilitates rapid conversion of polysulfides and regeneration of vacant active sites. Additionally, the catalyst must weaken internal molecular bonds and promote the surface diffusion of polysulfides.

#### Electrocatalytic materials for room-temperature Na||S batteries

**Fig. 4:**
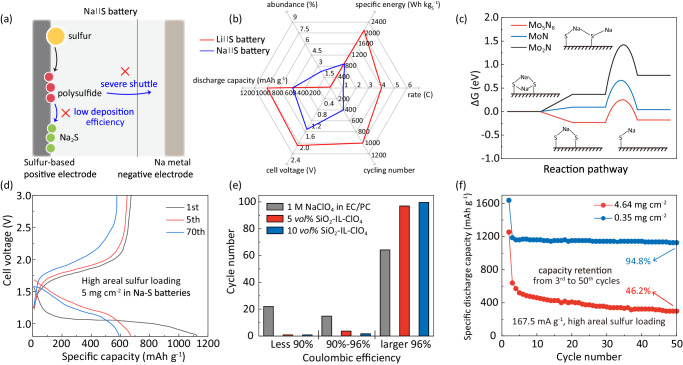
Electrocatalytic materials for room-temperature Na||S batteries. **a** Schematic showing the challenges for Na||S batteries including shuttle effect and low efficiency of Na_2_S deposition. **b** Schematic comparing the performance metrics of Li||S and Na||S batteries (Supplementary Table 2); **c** Reaction pathways for polysulfide conversion on Mo_5_N_6_, MoN and Mo_2_N in Na||S batteries. Reproduced with permission from ref. ^34^. Released under a Creative Commons Attribution 4.0 International License (https://creativecommons.org/licenses/by/4.0/). **d** Charge-discharge curves for MoN-catalyzed Na||S batteries with a high areal S loading. Adapted from Li et al. © 2022 Wiley‐VCH GmbH^35^. **e** Stability of homogenous additives in electrolytes during cycling. Reproduced with permission from ref. ^36^. Released under a Creative Commons Attribution 4.0 International License (https://creativecommons.org/licenses/by/4.0/). The electrolyte solution contained 1 M sodium perchlorate (NaClO_4_) in solvent of ethylene carbonate (EC) and propylene carbonate (PC) and an additive of 1-methyl-3-propylimidazolium-chlorate ionic liquid tethered silica nanoparticle (SiO_2_-IL-ClO_4_). **f** Cycling performance for Na||S batteries in presence of InI_3_ homogenous electrolyte additives with high areal S loading. Reproduced with permission from ref. ^37^. Released under a Creative Commons Attribution 4.0 International License (https://creativecommons.org/licenses/by/4.0/).

Na-S batteries represent the most extensively studied post-Li M||S batteries due to their promising high theoretical device-level specific energy for practical application in large-scale energy storage^3,4^. However, Na||S batteries face significant challenges, notably the low deposition efficiency of Na_2_S and the polysulfides shuttle effect, as depicted by the blue arrows in Fig. 4a. While Na||S batteries benefit from the higher abundance of sodium compared to the Li||S batteries, they suffer from lower specific capacity, shorter cycling life and lower charge-discharge rates (Fig. 4b and Supplementary Table 2). Researchers have explored heterogeneous and homogeneous electrocatalysts to mitigate these challenges in Na||S batteries. Ye et al., for example, reported a comparative study on electrocatalytic activity of selected metal nitrides, Mo_5_N_6_, Mo_2_N and MoN^34^ and found that Mo_5_N_6_ effectively reduced the free energy of polysulfide deposition, as depicted in Fig. 4c. The Mo_5_N_6_ catalyst enhances the Na_2_S electrodeposition, leading to increased specific capacity. The low deposition efficiency of Na_2_S is significant with high sulfur loading. To address this concern, Li et al., for example, reported use of a MoN catalyst to enhance Na_2_S electrodeposition and polysulfide conversion, resulting in an areal capacity of 2.5 mAh cm^−2^ under a sulfur loading of 5 mg cm^−2^, Fig. 4d^35^. In addition, the severe shuttle effect of polysulfides caused by the high solubility in the Na||S battery electrolyte is another significant challenge. Wei et al. mitigated this issue by introducing a SiO_2_-ionic liquid-ClO_4_ electrolyte additive in Na||S batteries^36^. The SiO_2_-ionic liquid functioned as a homogenous catalyst in the carbonate non-aqueous electrolyte solution, simultaneously enhancing S conversion kinetics and maintaining adequate electrochemical stability, Fig. 4e. The shuttle of sodium polysulfide was effectively restrained as evidenced by the improved CE. To further mitigate the shuttle effect and enhance the cyclic stability of Na||S batteries, Xu et al. employed an InI_3_ catalyst dispersed in the electrolyte solution to promote S conversion^37^. As is seen in Fig. 4f, the Na||S batteries utilizing the InI_3_ catalyst maintained a high capacity retention of 94.8% from 3 to 50 cycles with a low sulfur loading of 0.35 mg cm^−2^ at a 0.1 C (167.5 mA g^−1^) rate. Even with a high sulfur loading of 4.64 mg cm^−2^, the battery can still retain 46.2% specific capacity, indicating improved cyclic stability by using a homogeneous catalyst.

#### Electrocatalytic materials for room-temperature K||S, Mg||S, Ca||S and Al||S batteries

**Fig. 5:**
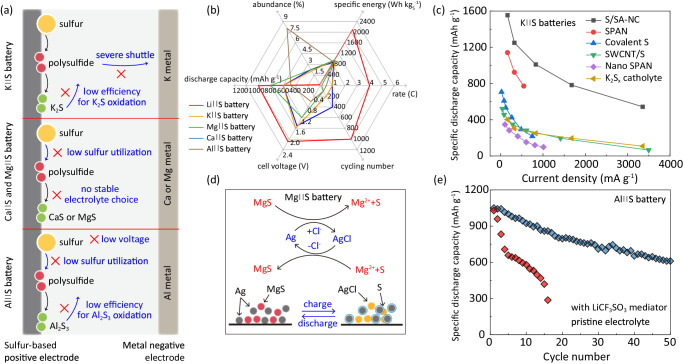
Electrocatalytic materials for room-temperature K||S, Mg||S, Ca||S and Al||S batteries. **a** Schematic showing the challenges for K||S, Ca||S, Mg||S and Al||S batteries. **b** Schematic comparing the performance metrics between Li||S and K||S, Ca||S, Mg||S, Al||S batteries (Supplementary Table 2). **c** Rate performance for SA-NC electrocatalyst in K||S battery at 25 °C compared with reported values. Adapted with permission from Ye et al. Copyright 2021 American Chemical Society^38^. **d** Schematic for S reaction in Mg||S battery with Ag catalyst. Reprint from Xu et al. with permission from Elsevier^39^. **e** Cycling performance for Al||S battery with a current rate of 0.05 C (83.75 mA g^−1^) at 25 °C with and without LiCF_3_SO_3_ mediator in electrolyte. Reprint from, Yu et al. with permission from Elsevier^14^.

K||S, Mg||S, Ca||S and Al||S, batteries represent emerging types of post-Li M||S batteries. Compared to Li||S and Na||S batteries, these configurations have received less attention in research, likely due to the practical challenges limiting their applications. As shown in Fig. 5a, for K||S batteries, the low efficiency for K_2_S oxidation and severe shuttle effect are the major concerns. For Mg/Ca||S batteries, the absence of a stable electrolyte option hinders sulfur utilization, resulting in low sulfur utilization and specific capacity. The Al||S batteries suffer from low discharge cell voltage due to the high electrochemical potential of the Al metal, along with low sulfur utilization and inefficient Al_2_S_3_ oxidation. Despite these challenges, each type of these post-Li M||S batteries offers distinct advantages that warrant further investigation for practical applications. For example, most K||S batteries exhibit a relative high discharge cell voltage (>1.5 V). The Mg/Ca||S batteries demonstrate balanced performance metrics. Al||S batteries possess the highest abundance (Fig. 5b and Supplementary Table 2). To address the concerns of these batteries, various catalysts have been proposed. Ye et al. introduced a single-atom catalyst (SA-NC, Co single atoms immobilized on N-doped carbon) for K||S batteries, Fig. 5c^38^. This catalyst enhanced oxidation of K_2_S, resulting in a high-rate capacity of 535 mAh g^−1^ under 3350 mA g^−1^ at room temperature. Compared with other K||S battery catalysts reported in the literature, it demonstrates the delivery of satisfactory discharge capacities at a high specific current. In Mg||S batteries, Xu et al. employed a Ag catalyst to enhance S conversion, coupled with a Cl^-^ containing MgCl_2_-YCl_3_ electrolyte^39^. The Ag foam catalyzed conversion from S to MgS via forming a AgCl layer on the Ag surface, achieving 100 stable cycles at 25 °C, Fig. 5d. However, most reported Mg||S batteries are tested under relatively low rates of ≤0.1 C (167.5 mA g^−1^)^21^. Yu et al. reported employing the lithium triflate (LiCF_3_SO_3_) as the homogeneous catalyst in Ca||S battery electrolyte, revealing that the Li ions reactivated Ca-based polysulfides by limiting the formation of strong Ca-S bonding, enabling the reversible operation of the Ca||S battery^40^. Similarly, they also reported a room-temperature Al||S battery with 0.5 M LiCF_3_SO_3_ as a homogeneous catalyst in the aluminum ionic liquid electrolyte to improve sulfur utilization through forming a Li_3_AlS_3_-like species, maintaining a discharge specific capacity of 600 mAh g^−1^ after 50 cycles at 0.05 C (83.75 mA g^−1^) under room temperature, Fig. 5e^14^.

#### Electrocatalytic materials in aqueous Zn||S batteries

**Fig. 6:**
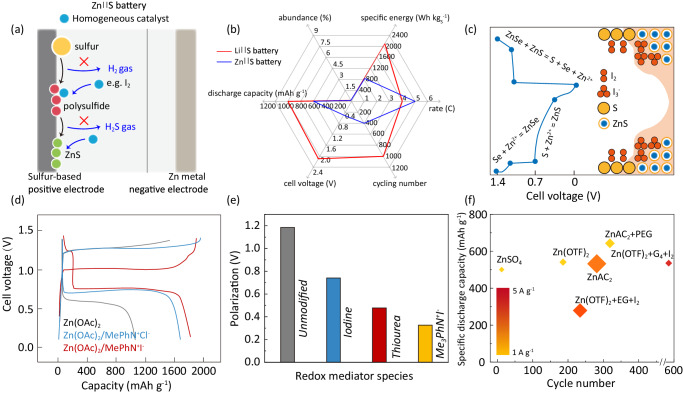
Electrocatalytic materials for aqueous Zn||S battery. **a** Schematic illustrating challenges and the homogeneous catalysts for Zn||S batteries^41,42^. **b** Schematic of the Li||S and Zn||S batteries’ performance metrics (Supplementary Table 2). **c** Mechanism of an I_2_-based homogeneous catalyst. Adapted with permission from Liu et al. Copyright 2023 American Chemical Society^43^. **d** Effect of cathodic catalysis on conversion from S to ZnS tested under 0.1 A g^−1^. Use with permission of The Royal Society of Chemistry, from Wu et al.; permission conveyed through Copyright Clearance Center, Inc^45^. **e** Reduced polarization induced by the dual mediators. The batteries were tested with a sulfur content of 26.4 *wt*.% and 2 M zinc sulfate (ZnSO_4_) aqueous electrolyte solution with various additives. The batteries were scanned from 0.2 V to 1.7 V with a scan rate of 0.2 mV s^−1^. Use with permission of The Royal Society of Chemistry, from Wu et al.; permission conveyed through Copyright Clearance Center, Inc^45^. Reprint from Chem et al., with permission from Elsevier^41^. **f** Comparative analysis of cycling performance with reported electrolytes and additives with S mass loading from 1.13 to 4 mg cm^−1^ in which, larger size refers to greater S loading mass. Reproduced with permission from refs. ^41,44,46–49^. All of the batteries were tested at room temperature. The testing conditions are 1 A g^−1^ with 1 M ZnSO_4_ in the electrolyte solution from ref. ^41^, 2 A g^−1^ with 1 M zinc acetate (ZnAC_2_) in the electrolyte solution from ref. ^41^, 1 A g^−1^ with 3 M zinc trifluoromethanesulfonate (Zn(OTF)_2_) in the electrolyte solution, 1 A g^−1^ with 1 M ZnAC_2_ and poly(ethylene glycol) in the electrolyte solution, 4 A g^−1^ with 2 M Zn(OTF)_2_, tetraglyme, and I_2_ in the electrolyte solution and 3 A g^−1^ with 2 M Zn(OTF)_2_, ethylene glycol and I_2_ in the electrolyte solution from refs. ^43–46^.

Aqueous Zn||S batteries face several challenges, including sluggish solid-solid conversion kinetics leading to voltage polarization higher than 0.2 V, deviating from the theoretical thermodynamic voltage value^25^. This aspect is associated with generation of H_2_S by-products and the formation of electrochemically inactive ZnS^25^. These factors contribute to lower the cell discharge voltage, specific energy and lifespan of aqueous Zn||S batteries, Fig. 6a, b. To address these challenges, researchers have explored the efficacy of homogeneous electrocatalysts in the electrolyte, which have shown promise in boosting polysulfide reduction, Fig. 6c^41–43^. Li et al. introduced iodine to mitigate the polarization of Zn||S batteries, reducing it from 1.0 to 0.72 V^44^. Figure 6d shows the effect of trimethylphenyl ammonium iodide (Me_3_PhN^+^I^−^), in which Me_3_PhN^+^ serves as a dissolution mediator and I^−^/I_3_^−^ as a redox mediator. The resultant S electrode exhibits 1659 mAh g^−1^ capacity with only 0.34 V overpotential at 0.1 A g^−1^ ^45^. These efforts have resulted in a significant reduction in polarization of Zn||S batteries to approximately <0.4 V, Fig. 6e^41,45^. Among these electrolytes, Zhu and co-workers developed a hybrid electrolyte solutions containing tetraglyme and water as solvents, iodine as additive, and Zn(OTF)_2_ salt, which inhibit generation of by-products and promote a uniform distribution of discharge product, delivering a discharge capacity of 496 mAh g^−1^ with more than after 600 cycles at 4 A g^−1^, Fig. 6f^38,44,46–49^.

### Challenges and outlooks

#### Electrolyte solutions and separators

The performance of sulfur electrodes and negative electrodes in the post-Li M||S batteries is significantly influenced by the characteristics of the electrolyte solutions^50^. The cations, the chain lengths of the polysulfides and the electrolyte solutions including solvents and salts play a crucial role in determining the solubility of the polysulfide species^51^.

**Fig. 7:**
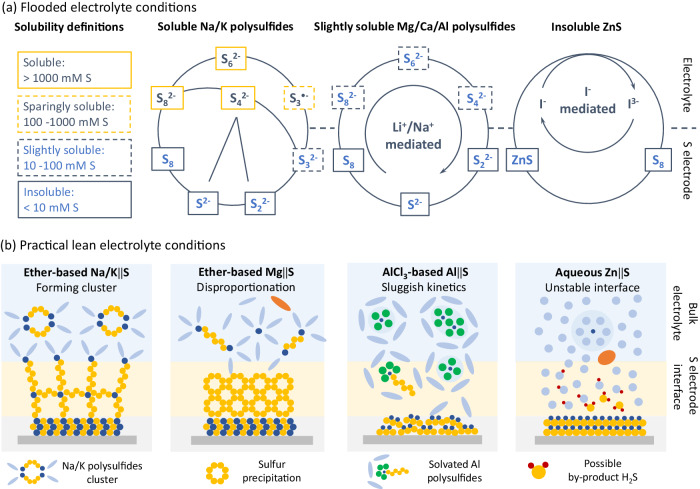
Sulfur reaction pathways in flooded and lean electrolyte conditions. **a** S reaction pathway and intermediates in the post-Li M||S batteries based on solubility. **b** Practical challenges under lean electrolyte. The orange-colored ovals, the light blue-colored ovals, the green spheres, the light blue-colored spheres, and the dark blue-colored spheres, and the red-colored spheres represent salt anions, solvent molecules, chloride anions, water molecules, cations and hydrogen ions presented in the electrolyte solution, respectively.

The solubility of sulfur species is defined as soluble (>1000 mM S), sparingly soluble (100–1000 mM S), slightly soluble (10–100 mM S) and insoluble (<10 mM S), Table 1. Accordingly, the reaction pathways and kinetic behavior in S electrochemical conversion in the M||S systems are illustrated in Fig. 7a. In Na/K||S batteries, in ether-based electrolytes such as tetraglyme, most of the polysulfides (S_4_^2−^, S_6_^2−^ and S_8_^2−^) are soluble, Table 1 Ref. ^52,53^. Under a flooded electrolyte condition, the catalytic pathway containing the S_3_^•-^ intermediate is mediated by the breaking of S-S bonds in the polysulfides, facilitating S redox processes^54^. It is noteworthy that due to the large size and low Lewis acidity of K ions, the solubility with S_3_^2-^ (~ 10.8 mM S) in dimethoxyethane is significantly lower than its counterparts of lithium and sodium electrolytes^53^. In Mg/Ca/Al||S batteries, electrolyte solutions commonly contain ethereal solvents, chloride, and borohydride anions^6^. For instance, the Mg and Ca salts in the ether-based electrolyte coupled with weakly coordinating anion borate such as Mg[B(hfip)_4_]_2_/Ca[B(hfip)_4_]_2_ demonstrate compatibility with the Mg/Ca metal negative electrode, in which the solubilities of long-chain polysulfides are lower than 50 mM S^55^. In Al||S systems, electrolytes facilitating reversible Al electrodeposition typically consist of a mixture of AlCl_3_ and organic salts chloride^56^. In AlCl_3_-based electrolytes, solubilities of the polysulfides is generally lower than 100 mM S^57^. The addition of Li/Na salts such as 0.5 M LiCF_3_SO_3_ can accelerate conversion kinetics and achieve an initial discharge capacity of 1000 mAh g^−1^ under 83.75 mA g^−1^ ^14^. In aqueous Zn||S systems, near-neutral aqueous-based electrolyte solutions are applied, where discharge product ZnS is insoluble (<10 mM S, Table 1), leading to large overpotentials of S conversion. To improve the conversion kinetics, the use of iodine species as redox mediators has been extensively studied^44^.

**Table 1:** Solubility of S_8_ and S_x_^2−^ (mM S) in the selected electrolyte solutions and solvents

Solutions/solvents	S_8_	S_8_^2−^	S_6_^2−^	S_4_^2−^	S_3_^2−^	S_2_^2−^	S^2−^	Refs.
0.98 M LiTFSI^a^ in TEGDME^b^	4	6046	–	34.1	–	13.9	0.8	^100^
Li_2_S_x_ in THF^c^	–	~10,000	~6500	~600	~300	~100	–	^101^
Li_2_S_x_ in DMSO^d^	3.9	~14,250	~6000	~1500	~800	~300	<0.2	^50^
Na_2_S_x_ in TEGDME	–	10,560	7140	2400	39	–	<6.7	^52^
K_2_S_x_ in DME^e^	–	–	–	–	~10.8	~1.6	–	^53^
1.0 M MgTFSI_2_ and 2.0 M MgCl_2_ in DME	9.6	<50	13.3	–	–	–	–	^57^
AlCl_3_ with EMIC^f^ in a molar ratio of 1.3:1	11.9	–	18.0	–	–	–	21.1	^56^

The solubilities of sulfur species are determined at room temperature unless otherwise specified.

^a^TFSI—bis(trifluoromethanesulfonyl) amide.

^b^TEGDME—teraglyme.

^c^THF—tetrahydrofuran.

^d^DMSO—dimethyl sulfoxide.

^e^DME—dimethyl ether.

^f^EMIC − 1-ethyl-3-methylimidazoliumchloride.

It is well-established that the Li||S battery performance is closely related to the lean electrolyte conditions (under the R_E/S_ of 4 μL mg^−1^)^58,59^. However, this is rarely discussed in the post-Li M||S batteries. In Na/K||S batteries under the lean electrolyte environment, based on the hard-soft acid-base theory, the hard-acid Na/K ions preferentially coordinate with the highly soluble polysulfide dianions to form gel-like solid polysulfides, which hinders the Na/K ion diffusion and impedes S redox kinetic, Fig. 7b^58^. For Mg/Ca||S batteries, strong interactions between Mg and polysulfides lead to disproportionation of long-chain Mg polysulfides into S and short-chain Mg polysulfide species, which becomes more abundant in lean electrolyte condition^60,61^. In Al||S batteries, the sluggish S solid-state conversion kinetics arise from the poor electrochemical activities of the sulfur species and high dissociation energy of Al ions from the electrolyte. In aqueous Zn||S batteries, the Pourbaix diagram illustrates the electrochemical conversion governed by pH and potential at 25 °C. At potentials lower than 0 V, the S^2−^ (pH >13.9), HS^−^ (pH = 7–13.9), H_2_S (pH <7) are the main sulfur species^25^. Under lean-electrolyte conditions, such as in 2 M ZnSO_4_ with a pH of ~3.8, a significant pH change and an inhomogeneous pH distribution at sulfur electrode|electrolyte interface can lead to severe side reactions such as H_2_S evolution, depleting the aqueous-based electrolyte solutions.

Inspired by Li||S batteries, regulating electrolyte solutions to enable quasi-solid-state S redox reaction is expected to mitigate the shuttle effect and polysulfide gelation in Na/K||S batteries^62,63^. It has been reported that by incorporating fluorinated co-solvents such as 1,1,2,2-tetrafluoroethyl 2,2,3,3-tetrafluoropropyl ether or utilizing solvent-in-salt electrolytes, the induced quasi-solid-state S conversion enhances the cycling stability of S electrodes^64,65^. This approach may increase the overpotential of S conversion, although the catalytic mechanism has been rarely investigated. For Mg/Ca||S batteries, adjusting electrolyte solution composition to weaken the Mg-polysulfides interaction is a promising strategy to improve stability of the polysulfides. However, this may lower the solubility of the Mg polysulfides, restricting Mg dissociation and S conversion^66^. Exploring alternative electrolyte systems such as molten salt electrolytes and operating at elevated temperature (85 °C) may improve kinetics of Al||S batteries^67^. Recently, Pang and co-workers developed a rapid-charging Al||S battery with a tamed quaternary molten salt electrolyte and operated at a sub-water-boiling temperature of 85 °C, demonstrating 85.4% capacity retention over 1400 cycles under 1675 mA g^−1^ ^67^. In Zn||S batteries, adding co-solvents such as the tetraglyme or ethylene glycol and employing gel polymer electrolytes can help mitigate the kinetic challenges^6,49,68–70^.

Employing functional separators represents an effective strategy to improve the CE of the M||S batteries^71^. Inspired by the achievements in Li||S batteries, modifications of commercial thin separators can be implemented for ether-based electrolyte systems such as Na/K/Mg/Ca||S batteries^72,73^. For instance, by coupling a thin poly (vinyl alcohol) (PVA) separator with an interlayer consisting of graphene oxide and Cu nanoparticles, a Mg||S battery prototype with 53.4 Wh kg^−1^ was achieved^31^. However, the impact on device-level specific energy must be carefully considered. When heavy transition metals are incorporated, functional separators often exhibit increased thickness and electrolyte uptake compared to commercial separators, resulting in reduced device-level specific energy. An ideal industrial functional separator should have a mass loading lower than 0.1 mg cm^−2^ ^74^. The additional costs (associated with the use of additives for the functionalization, like carbon-based nanomaterials) and safety-related parameters including thermal resistance, flame retardancy and mechanical robustness also need to be considered. So far, the Zn/Al||S batteries suffer from the lack of commercially available thin separators suitable for the adoption of lean electrolyte conditions. Overall, the properties of a separator can profoundly affect the performance of the post-Li M||S battery. The impact of separators on negative electrodes is discussed in the “Post-lithium metal negative electrodes” section.

#### Sulfur-based positive electrodes

In room-temperature Na||S batteries, the dissociation of long-chain polysulfides is a significant practical challenge, and therefore, it is important to boost stability of long-chain polysulfides in the electrolyte together with conversion kinetics. In K||S and Zn||S batteries, the presence of high energy barriers in K_2_S and ZnS oxidation limits the reversibility and high-rate performance. Catalyst design aimed at facilitating K_2_S and ZnS decomposition during the charging process is important for long-cycled K||S and Zn||S batteries. In Al||S batteries, the Al-based metal ions (such as Al_x_Cl_y_^−^) present large ion radius, and therefore, the reactivity between these large ions and S/Al_2_S_3_ is restricted. The catalyst design should target both the sulfur reduction and Al_2_S_3_ oxidation reactions, that is, a bidirectional catalyst is preferred. Unlike other types of M||S batteries, there are no commonly used electrolytes in Ca||S and Mg||S batteries. Therefore, choice of electrolyte is of vital importance because Mg- and Ca-based polysulfides are not electrochemically stable in conventional ether- or ester-based electrolytes. To boost cyclic stability of Mg||S and Ca||S batteries, a combining employment of electrocatalyst and compatible electrolyte solutions may be promising.

In the design of heterogeneous catalysts, a balanced affinity between catalysts and polysulfide species is needed to facilitate surface charge transfer and subsequent polysulfide diffusion. Additionally, high electrical conductivity is required particularly in high-loading or high-rate conditions, otherwise high current density leads to large overpotentials deteriorating the cyclic stability and discharge capacity. The structure of the catalyst is important and needs to ensure full exposure of active sites and facilitate fast ion transport. An interconnected three-dimensional network structure is desirable as it allows for accommodation of high-loading S, maximizes use of active sites and promotes rapid ion diffusion throughout the catalyst. The chemical and electrochemical stability of catalysts is important in ensuring favorable long-term cycling performance of M||S batteries. The stability of catalysts in an active state is important in sustaining persistent kinetic promotion throughout battery operation.

For the homogeneous catalysts, good solubility and diffusivity in electrolyte are the prior concerns. These two parameters ensure fast transport of homogeneous catalysts to the electrode surface. After approaching the electrode surface, a sufficient reaction between these homogeneous catalysts and polysulfides is required, especially in high-S-loading configurations and under lean-electrolyte condition. This is determined by several factors, such as a suitable redox potential, electrochemical activity of catalysts, affinity and interactions between catalysts and polysulfides. Additionally, adequate electrochemical stability is necessary to sustain functionality of homogeneous catalysts and enable improved long-term cycling performance.

#### Post-lithium metal negative electrodes

A significant challenge encountered by metal negative electrodes is associated with the limited electrochemical cycling reversibility, which derives from their reaction with electrolyte solutions^75^. In Na/K metal negative electrodes, it is a practical strategy to finely tune the components of the electrolyte solutions to form robust and conductive SEIs. For instance, in K||S batteries, electrolytes with 3 M potassium bis(trifluoromethanesulfonyl)imide salt in ethylene carbonate have been employed to form a stable KF-rich SEI on the K metal surface, extending the lifespan of the K||S to 2000 cycles at 1 A g^−1^ ^64,76,77^. Another effective strategy involves coating the metal with protective films prior to contact with the electrolyte solutions^78^. Additionally, the employment of solid electrolytes such as Na_3_PS_4_ or Na_3_Zr_2_Si_2_PO_12_ filler in PEO-NaFSI (poly(ethylene oxide)-sodium bis(fluorosulfonyl) imide), or K_2.92_Sb_0.92_W_0.08_S_4_ has been explored^79–81^. It is noteworthy that the dissolution of polysulfide moieties poses a unique challenge to achieve high CE. Therefore, it is recommended to conduct the electrochemical cell testing both in the presence and absence of polysulfide species in the selected electrolyte solutions, followed by tests in lean electrolyte environments^75^. Finally, to reach the theoretical specific energy, it is essential to reduce the R_N/P_ to a practical level of approximately 1 utilizing thin Na/K negative electrodes^82,83^. In this regard, the investigations of thin Na/K metal and the anode-free configuration should be inspiring for developing high-specific-energy Na/K||S batteries. The anode-free configuration can be realized through constructing a robust and highly conductive SEI. This is related with optimizing the negative electrode current collectors’ surface properties and electrolyte additives^84,85^.

In the Mg/Ca||S batteries, a significant challenge arises from the passivation of the Mg/Ca negative electrodes by dissolved polysulfides, resulting from low ionic conductivities of the SEIs^86^. Even trace amounts of polysulfides in electrolyte solutions can lead to the precipitation of sulfur species, affecting the cycling reversibility of Mg and Ca metal negative electrodes^87,88^. Interestingly, dissolved iodine has been reported to improve the ionic conductivity of the SEIs^89^. Although the passivation issue is more severe for Ca metal compared to Mg metal, research in this area remains limited^31^. Therefore, validating the practicality of the Mg and Ca metal negative electrodes in Mg/Ca||S batteries requires comprehensive characterization of the metal negative electrode in electrolyte solutions containing polysulfides. Recent research has been focused on the application of thin Mg negative electrodes, while the related studies under the lean electrolyte environment are limited^90,91^.

To suppress Al dendrites formation and achieve high CE, strategies on Al structure design such as 3D structure and amorphization have been employed^92^. However, most reported Al metal negative electrodes are tested under low-capacity utilization. For instance, commercial Al foils with thickness of approximately 0.1 mm possesses a theoretical areal capacity of ~75 mAh cm^−2^, while most reports cycled at the depth of ~0.3 mAh cm^−2^ ^93^. It is essential to consider this condition because the growth of Al dendrites at high areal capacity significantly affects the CE and stability of batteries. Recently, Chen and colleagues developed an Au-modified Ti foil as the current collector for anode-free Al metal, revealing that the modified current collector enhanced Al nucleation density and reduced average particle size of Al deposits. This results in highly reversible Al cycling process with high CE of 99.92%^93^. Another challenge for the Al metal negative electrode is corrosion by electrolyte solutions, which hinders realization of a high CE above 99.9%. Effective strategy to suppress this include tuning components of the electrolyte, adding anti-corrosive additives and manipulating surface properties of Al metal^92^.

In aqueous Zn||S batteries, similar challenges arise from hydrogen evolution reaction, a phenomenon persistent throughout the battery’s operational and rest cycles. Unlike Mg/Ca/Al ions, Zn ions exhibit smoother solid-state diffusion in inorganic hosts, possibly attributed to their chemical nature as ‘borderline’ cations in the hard-soft acid-base theory, forming more-covalent bonds with host anions^94^. Based on this property, various strategies such as constructing robust and conductive SEIs or solid-state electrolytes have been developed^43,68^. Additionally, most reported Zn metal negative electrodes are tested under conditions of low-capacity utilization and flood electrolyte environments^95,96^.

The post-Li M||S batteries may share the successful operating mechanism with Li||S batteries while using more abundant elements as ionic charge carriers. Calculations show that these batteries may deliver acceptable specific energies for large-scale energy storage. From this aspect, cycling stability and safety are critical for the post-Li M||S batteries. Although there are attempts to employ alloying materials as negative electrodes, this results in lowering specific energies because of their relatively low-capacity and high-potential^89,97,98^. To improve the CE and safety of the Na/K/Zn||S batteries, developing solid-state electrolytes is promising because of their cations’ acceptable conductivities as discussed in the last section. However, achieving quasi-solid-state or solid-state conversion in Mg/Ca/Al||S batteries is challenging due to the high charge densities of multivalent cations, making dissociation and solid-state diffusion kinetically unfavorable. Instead, it is a practical approach to develop liquid-based electrolytes such as molten salts under high-temperature operation. However, it is important to consider the potential safety hazard associated with high-temperature operation (e.g., those observed in high-temperature Na||S batteries), as well as the challenges posed by corrosive environments (e.g., those observed in Al||S batteries)^27,99^. For both cases, the low CE and sluggish conversion kinetics of the sulfur species in sulfur electrodes interfaces remains a primary challenge for post-Li M||S batteries development. Synergy of heterogeneous catalysts in the sulfur cathodes, homogeneous catalysts in the electrolyte and electrolyte solutions design is promising to solve this kinetical challenge. More importantly, the design of the electrocatalytic materials and the investigation of the conversion mechanisms need to consider challenges including sulfur electrodes with high sulfur loading/content, electrochemical/mechanical stability at the sulfur electrode|electrolyte interface, electrode/cell design under elevated operation temperatures, etc. Although the research of post-Li M||S batteries is still at an early stage with many challenges to be addressed, we anticipate that the intense academic interest and the encouraging recent progress are forging a clear path for their widespread application.

## Supplementary information

Supplementary Information
