# Supplementary material for: The role of electrocatalytic materials for developing post-lithium metal||sulfur batteries
Source: Nat Commun. 2024 Jun 5;15:4797. doi: 10.1038/s41467-024-49164-6 (PMC11535197; doi:10.1038/s41467-024-49164-6)
Supplement: Supplementary file 1 — Supplementary Information [file 41467_2024_49164_MOESM1_ESM.pdf]

The role of electrocatalytic materials for developing post-lithium metal||sulfur batteries

Chao Ye[1]†, Huan Li[1]†, Yujie Chen[1], Junnan Hao[1], Jiahao Liu[1], Jieqiong Shan[2], Shi-Zhang Qiao[1,*]

[1] School of Chemical Engineering, The University of Adelaide, Adelaide, SA 5005, Australia.

[2] Department of Chemistry, City University of Hong Kong, Kowloon, Hong Kong 999077, P.R. China

† These authors contributed equally to this work.

*Email: s.qiao@adelaide.edu.au

Supplementary Tables

Supplementary Table 1. The acronyms and associated full names in **Figure 1a-1d**.

Systems	Acronyms	Full names	Ref
Na/K‖S	S-PAA-4M KTFSI	Sulfur-based positive electrode containing poly (acrylic acid) with 5M Potassium bis (trifluoromethane sulphonyl) imide electrolyte	1
	S-CMK3-5M KTFSI	Sulfur in ordered mesoporous carbon CMK-3 with 5M Potassium bis (trifluoromethane sulphonyl) imide electrolyte	2
	Polysulfide CN	Polysulfide in carbon nanotubes	3
	S-MC	Sulfur in microporous carbon	4
	S-CNF-SWCNT	Sulfur in carbon nanofiber with a single-wall carbon nanotube coated separator	5
	S-MPCF	Sulfur in multiporous carbon fibers	6
	SPAN	Sulfurized polyacrylonitrile	7
	S-CuSA/NC	Sulfur in nitrogen-doped carbon with Cu single-atom catalyst	8
	SPAN-PAA	Sulfurized polyacrylonitrile with poly (acrylic acid) binder	9
	S-Mo_2N-W_2N	Sulfur-based electrode with a Mo_2N-W_2N heterostructure catalyst	10
	S-Ni-MOF	Sulfur-based electrode with a Ni-based metal-organic framework additive	11
	S-Ni-NCF	Sulfur in nitrogen-doped carbon fibers with a Ni hollow sphere catalyst	12
	S-MCHS	Sulfur in mesoporous carbon hollow nanospheres	13
Mg/Ca‖S	S-ACC	Sulfur in activated carbon cloth	14
	S-KB	Sulfur in Ketjenblack	15
	S-ACC	Sulfur in activated carbon cloth	16
	S-C	sulfur in porous carbon	17
	S-ZIF-C	Sulfur in zeolitic imidazolate frameworks-67 derivative carbon host	18
	S-rGO-CC	Sulfur with reduced graphene oxide in carbon cloth	19
	S-Cu-CFs	Sulfur in carbon nanofibers with a Cu nanoparticle additive	20
	S-MC	Sulfur in microporous carbon	21
	S-meso-C	Sulfur in mesoporous carbon	22
	S-G-CC	Sulfur in graphene-polyaniline coated carbon cloth	23
	S-CNT	Sulfur in carbon nanotube	24
	S-KB-DMSO	Sulfur in Ketjenblack with dimethyl sulfoxide-based electrolyte solutions	25
Al‖S	S-MWCNT	Sulfur in multi-wall carbon nanotube	26

	S-HPC	Sulfur in hierarchical porous carbon	27
	S-CNF	Sulfur in carbon nanofiber	28
	S-CoNG	Sulfur in cobalt/nitrogen co-doped graphene	29
	S-KB	Sulfur in Ketjenblack	30
	S-HPC	Sulfur in N-doped hierarchical porous carbon	31
	S-CNT	Sulfur in carbon nanotube	32
	S-CNT	Sulfur in carbon nanotube	33
	S-KB	Sulfur in Ketjenblack	34
	S-CNF	Sulfur in carbon nanofiber paper	35
	S-CNT	Sulfur in carbon nanotube	36
Zn‖S	S-AC-I_2	Sulfur in activated carbon with the I_2 additive in the electrolyte	37
	ZnS-CF-TU	ZnS fiber with the iodinated thiourea additive in the electrolyte solution	38
	S-NPC-ZnI_2	Sulfur in nanoporous carbon with the zinc iodide additive in the electrolyte solution	39
	S-CMK3-ZnI_2	Sulfur in ordered mesoporous carbon CMK-3 with the zinc iodide additive in the electrolyte solution	40
	S-KB-TU	Sulfur in Ketjenblack with the thiourea additive in the electrolyte solution	41
	S-Se	Sulfur mixed with selenium as active materials in the positive electrode	42
	S-C-AN	Sulfur in carbon black with the acetonitrile additive in the electrolyte solution	43
	S-CNT-PEG	Sulfur in multi-walled carbon nanotubes with the polyethylene glycol additive in the electrolyte solution	44
	S-CNT-I_2	Sulfur in multi-walled carbon nanotubes with the iodine additive in the electrolyte solution	45
	SeS_2-PCS-I_2	SeS_2 in phosphorus-doped carbon sheets with the iodine additive in the electrolyte solution	46
	S-FeNC-CC	Sulfur in carbon cloth with the Fe-N_4 catalyst	47
	S-HCS- I_2	Sulfur in hollow carbon spheres with the iodine additive in the electrolyte solution	48
	S-CNF- I_2	Sulfur in carbon nanofibers with the iodine additive in the electrolyte solution	49
	S-CMK3	Sulfur in ordered mesoporous carbon CMK-3	50

Supplementary Table 2. Comparison for different metal-sulfur batteries in terms of earth abundance of metals, capacity based on sulfur electrode, cell voltage, cycling number, rate (1C = 1675 mA g^{-1}), specific energy (based on sulfur electrode), sulfur content and areal sulfur loading. The values are estimated by averaging those from a couple of representative references. The values are all tested under the room temperature or 25 ^{o}C.

	Abundance / % among earth crust[51]	Capacity /mAh g^{-1}	Cell voltage/ V	Cycling number	Rate/ C	Specific energy/ Wh kg_S^{-1}	Sulfur content/ *wt.*%	Areal sulfur loading/ mg cm^{-2}	Ref
Li||S	0.002	≈1000	≈2.1	≈1000	3	≈2100	≈50-72	≈5	52-56
Na||S	2.36	≈600	≈1.5	≈800	1	≈900	≈40-60	≈3-5	57-59
K||S	2.09	≈600	≈1.6	≈200	1	≈960	≈50	≈1-2	60
Mg||S	2.33	≈800	≈1.2	≈100	0.1	≈960	≈30-70	≈1	61, 62
Ca||S	4.15	≈800	≈1.1	≈50	0.1	≈960	≈30-50	≈0.5-2	63, 64
Al||S	8.23	≈500	≈0.6	≈100	0.5	≈900	≈40-70	≈0.5-2	35, 65, 66
Zn||S	0.007	≈600	≈0.5	≈400	5	≈900	≈40-70	≈1-2	41, 67, 68

Supplementary References

1. Yang K, *et al.* Achieving Fast and Reversible Sulfur Redox by Proper Interaction of Electrolyte in Potassium Batteries. *ACS Energy Lett* **8**, 2169-2176 (2023).

2. Wang L, Bao J, Liu Q, Sun C-F. Concentrated electrolytes unlock the full energy potential of potassium-sulfur battery chemistry. *Energy Storage Mater* **18**, 470-475 (2018).

3. Gu S, Xiao N, Wu F, Bai Y, Wu C, Wu Y. Chemical Synthesis of K2S2 and K2S3 for Probing Electrochemical Mechanisms in K–S Batteries. *ACS Energy Lett* **3**, 2858-2864 (2018).

4. Zhao X, *et al.* High performance potassium–sulfur batteries and their reaction mechanism. *J Mater Chem A* **8**, 10875-10884 (2020).

5. Yu X, Manthiram A. A Reversible Nonaqueous Room-temperature Potassium-Sulfur Chemistry for Electrochemical Energy Storage. *Energy Storage Mater* **15**, 368-373 (2018).

6. Xu X, *et al.* A room-temperature sodium-sulfur battery with high capacity and stable cycling performance. *Nat Commun* **9**, 3870 (2018).

7. Wang J, Yang J, Nuli Y, Holze R. Room temperature Na/S batteries with sulfur composite cathode materials. *Electrochem Commun* **9**, 31-34 (2007).

8. Xiao F, *et al.* Generating Short-Chain Sulfur Suitable for Efficient Sodium–Sulfur Batteries via Atomic Copper Sites on a N,O-Codoped Carbon Composite. *Adv Energy Mater* **11**, 2100989 (2021).

9. Hwang J-Y, Kim HM, Sun Y-K. High performance potassium–sulfur batteries based on a sulfurized polyacrylonitrile cathode and polyacrylic acid binder. *J Mater Chem A* **6**, 14587-14593 (2018).

10. Zhang S, *et al.* Mo2N–W2N Heterostructures Embedded in Spherical Carbon Superstructure as Highly Efficient Polysulfide Electrocatalysts for Stable Room-Temperature Na–S Batteries. *Adv Mater* **33**, 2103846 (2021).

11. Ye C, *et al.* Electron-State Confinement of Polysulfides for Highly Stable Sodium–Sulfur Batteries. *Adv Mater* **32**, 1907557 (2020).

12. Guo B, *et al.* Nickel Hollow Spheres Concatenated by Nitrogen-Doped Carbon Fibers for Enhancing Electrochemical Kinetics of Sodium–Sulfur Batteries. *Adv Sci* **7**, 1902617 (2020).

13. Wang Y-X, *et al.* Achieving high-performance room-temperature sodium-sulfur batteries with S@interconnected mesoporous carbon hollow nanospheres. *J Am Chem Soc* **138**, 16576-16579 (2016).

14. Scafuri A*, et al.* Spectroscopic Insights into the Electrochemical Mechanism of Rechargeable Calcium/Sulfur Batteries. *Chem Mater* **32**, 8266-8275 (2020).

15. Li Z, Vinayan BP, Diemant T, Behm RJ, Fichtner M, Zhao-Karger Z. Rechargeable Calcium–Sulfur Batteries Enabled by an Efficient Borate-Based Electrolyte. *Small* **16**, 2001806 (2020).

16. Zhao-Karger Z*, et al.* Toward highly reversible magnesium–sulfur batteries with efficient and practical Mg [B (hfip) 4] 2 electrolyte. *ACS Energy Lett* **3**, 2005-2013 (2018).

17. Zhou D*, et al.* Multi-ion Strategy toward Highly Durable Calcium/Sodium–Sulfur Hybrid Battery. *Nano Lett* **21**, 3548-3556 (2021).

18. Zhou X, Tian J, Hu J, Li C. High Rate Magnesium–Sulfur Battery with Improved Cyclability Based on Metal–Organic Framework Derivative Carbon Host. *Adv Mater* **30**, 1704166 (2018).

19. Muthuraj D, Ghosh A, Kumar A, Mitra S. Nitrogen and Sulfur Doped Carbon Cloth as Current Collector and Polysulfide Immobilizer for Magnesium-Sulfur Batteries. *ChemElectroChem* **6**, 684-689 (2019).

20. He P, Ford HO, Merrill LC, Schaefer JL. Investigation of the Effects of Copper Nanoparticles on Magnesium–Sulfur Battery Performance: How Practical Is Metallic Copper Addition? *ACS Appl Energy Mater* **2**, 6800-6807 (2019).

21. Wang W, Yuan H, NuLi Y, Zhou J, Yang J, Wang J. Sulfur@microporous Carbon Cathode with a High Sulfur Content for Magnesium–Sulfur Batteries with Nucleophilic Electrolytes. *J Phys Chem C* **122**, 26764-26776 (2018).

22. Zhang Z*, et al.* Novel Design Concepts of Efficient Mg-Ion Electrolytes toward High-Performance Magnesium–Selenium and Magnesium–Sulfur Batteries. *Adv Energy Mater* **7**, 1602055 (2017).

23. Bosubabu D, Li Z, Meng Z, Wang L-P, Fichtner M, Zhao-Karger Z. Mitigating self-discharge and improving the performance of Mg–S battery in Mg[B(hfip)4]2 electrolyte with a protective interlayer. *J Mater Chem A* **9**, 25150-25159 (2021).

24. Du A*, et al.* An efficient organic magnesium borate-based electrolyte with non-nucleophilic characteristics for magnesium–sulfur battery. *Energy Environ Sci* **10**, 2616-2625 (2017).

25. Zou Q, Sun Y, Liang Z, Wang W, Lu Y-C. Achieving Efficient Magnesium–Sulfur Battery Chemistry via Polysulfide Mediation. *Adv Energy Mater* **11**, 2101552 (2021).

26. Yang H*, et al.* An Aluminum–Sulfur Battery with a Fast Kinetic Response. *Angew Chem Int Ed* **57**, 1898-1902 (2018).

27. Zhang D, *et al.* High-Voltage Aluminium-Sulfur Batteries with Functional Polymer Membrane. *Adv Funct Mater* **32**, 2205562 (2022).

28. Yu X, Manthiram A. Electrochemical Energy Storage with a Reversible Nonaqueous Room-Temperature Aluminum–Sulfur Chemistry. *Adv Energy Mater* **7**, 1700561 (2017).

29. Huang Z, *et al.* Electrocatalysis for Continuous Multi-Step Reactions in Quasi-Solid-State Electrolytes Towards High-Energy and Long-Life Aluminum–Sulfur Batteries. *Angew Chem Int Ed* **61**, e202202696 (2022).

30. Cohn G, Ma L, Archer LA. A novel non-aqueous aluminum sulfur battery. *J Power Sources* **283**, 416-422 (2015).

31. Zhang D, *et al.* Highly reversible aluminium–sulfur batteries obtained through effective sulfur confinement with hierarchical porous carbon. *J Mater Chem A* **9**, 8966-8974 (2021).

32. Gao T, *et al.* A Rechargeable Al/S Battery with an Ionic-Liquid Electrolyte. *Angew Chem Int Ed* **55**, 9898-9901 (2016).

33. Li H, *et al.* Reversible electrochemical oxidation of sulfur in ionic liquid for high-voltage Al−S batteries. *Nat Commun* **12**, 5714 (2021).

34. Jay R, Jadhav AL, Gordon LW, Messinger RJ. Soluble Electrolyte-Coordinated Sulfide Species Revealed in Al–S Batteries by Nuclear Magnetic Resonance Spectroscopy. *Chem Mater* **34**, 4486-4495 (2022).

35. Yu X, Boyer MJ, Hwang GS, Manthiram A. Room-Temperature Aluminum-Sulfur Batteries with a Lithium-Ion-Mediated Ionic Liquid Electrolyte. *Chem* **4**, 586-598 (2018).

36. Li H, Lampkin J, Garcia-Araez N. Facilitating Charge Reactions in Al-S Batteries with Redox Mediators. *ChemSusChem* **14**, 3139-3146 (2021).

37. Amiri A, Bashandeh K, Sellers R, Vaught L, Naraghi M, Polycarpou AA. Fully integrated design of a stretchable kirigami-inspired micro-sized zinc–sulfur battery. *J Mater Chem A* **11**, 10788-10797 (2023).

38. Liu D, *et al.* A durable ZnS cathode for aqueous Zn-S batteries. *Nano Energy* **101**, 107474 (2022).

39. Guo Y, *et al.* Hybrid Electrolyte Design for High-Performance Zinc–Sulfur Battery. *Small* **19**, 2207133 (2023).

40. Li J, Cheng Z, Li Z, Huang Y. Rational design of zinc powder anode with high utilization and long cycle life for advanced aqueous Zn–S batteries. *Mater Horiz* **10**, 2436 (2023).

41. Chang G*, et al.* Bifunctional electrolyte additive with redox mediation and capacity contribution for sulfur cathode in aqueous Zn-S batteries. *Chem Eng J* **457**, 141083 (2023).

42. Liu J, Ye C, Wu H, Jaroniec M, Qiao S-Z. 2D Mesoporous Zincophilic Sieve for High-Rate Sulfur-Based Aqueous Zinc Batteries. *J Am Chem Soc* **145**, 5384–5392 (2023).

43. Cui M, Fei J, Mo F, Lei H, Huang Y. Ultra-High-Capacity and Dendrite-Free Zinc–Sulfur Conversion Batteries Based on a Low-Cost Deep Eutectic Solvent. *ACS Appl Mater Interfaces* **13**, 54981-54989 (2021).

44. Zhou T, Wan H, Liu M, Wu Q, Fan Z, Zhu Y. Regulating uniform nucleation of ZnS enables low-polarized and high stable aqueous Zn–S batteries. *Mater Today Energy* **27**, 101025 (2022).

45. Li W, Wang K, Jiang K. A Low Cost Aqueous Zn–S Battery Realizing Ultrahigh Energy Density. *Adv Sci* **7**, 2000761 (2020).

46. Li W*, et al.* Phosphorus-doped carbon sheets decorated with SeS2 as a cathode for aqueous Zn-SeS2 battery. *Chem Eng J* **420**, 129920 (2021).

47. Zhang W*, et al.* Bidirectional Atomic Iron Catalysis of Sulfur Redox Conversion in High-Energy Flexible Zn□S Battery. *Adv Funct Mater* **33**, 2210899 (2023).

48. Yang M*, et al.* Boosting Cathode Activity and Anode Stability of Zn-S Batteries in Aqueous Media Through Cosolvent-Catalyst Synergy. *Angew Chem Int Ed* **61**, e202212666 (2022).

49. Amiri A, Sellers R, Naraghi M, Polycarpou AA. Multifunctional Quasi-Solid-State Zinc–Sulfur Battery. *ACS Nano* **17**, 1217-1228 (2023).

50. Xu Z*, et al.* The key role of concentrated Zn(OTF)2 electrolyte in the performance of aqueous Zn–S batteries. *Chem Commun* **58**, 8145-8148 (2022).

51. Taylor SR. Abundance of chemical elements in the continental crust: a new table. *Geochim Cosmochim Acta* **28**, 1273-1285 (1964).

52. Zhao C*, et al.* A high-energy and long-cycling lithium–sulfur pouch cell via a macroporous catalytic cathode with double-end binding sites. *Nat Nanotechnol* **16**, 166-173 (2020).

53. Hua W*, et al.* Selective Catalysis Remedies Polysulfide Shuttling in Lithium-Sulfur Batteries. *Adv Mater* **33**, 2101006 (2021).

54. Yang Y, Zhong Y, Shi Q, Wang Z, Sun K, Wang H. Electrocatalysis in Lithium Sulfur Batteries under Lean Electrolyte Conditions. *Angew Chem Int Ed* **57**, 15549-15552 (2018).

55. Song Y, Cai W, Kong L, Cai J, Zhang Q, Sun J. Rationalizing Electrocatalysis of Li–S Chemistry by Mediator Design: Progress and Prospects. *Adv Energy Mater* **10**, 1901075 (2019).

56. Zhou G, Chen H, Cui Y. Formulating energy density for designing practical lithium–sulfur batteries. *Nat Energy* **7**, 312-319 (2022).

57. Li Z*, et al.* Room-Temperature Sodium-Sulfur Batteries: Rules for Catalyst Selection and Electrode Design. *Adv Mater* **34**, e2204214 (2022).

58. Zhang E*, et al.* Single-Atom Yttrium Engineering Janus Electrode for Rechargeable Na-S Batteries. *J Am Chem Soc* **144**, 18995-19007 (2022).

59. Xu X*, et al.* A room-temperature sodium-sulfur battery with high capacity and stable cycling performance. *Nat Commun* **9**, 3870 (2018).

60. Ye C*, et al.* Catalytic Oxidation of K2S via Atomic Co and Pyridinic N Synergy in Potassium-Sulfur Batteries. *J Am Chem Soc* **143**, 16902-16907 (2021).

61. Xu Y*, et al.* Reversible function switching of Ag catalyst in Mg/S battery with chloride-containing electrolyte. *Energy Storage Mater* **42**, 513-516 (2021).

62. Zhang Z*, et al.* Progress and prospects for solving the "shuttle effect" in magnesium-sulfur batteries. *Energy Storage Mater* **62**, 102933 (2023).

63. Yu X, Boyer MJ, Hwang GS, Manthiram A. Toward a Reversible Calcium-Sulfur Battery with a Lithium-Ion Mediation Approach. *Adv Energy Mater* **9**, 1803794 (2019).

64. Li Z, Vinayan BP, Diemant T, Behm RJ, Fichtner M, Zhao-Karger Z. Rechargeable Calcium-Sulfur Batteries Enabled by an Efficient Borate-Based Electrolyte. *Small* **16**, e2001806 (2020).

65. Guo Y*, et al.* Rechargeable Aluminium-Sulfur Battery with Improved Electrochemical Performance by Cobalt-Containing Electrocatalyst. *Angew Chem Int Ed* **59**, 22963-22967 (2020).

66. Huang Z*, et al.* Electrocatalysis for Continuous Multi-Step Reactions in Quasi-Solid-State Electrolytes Towards High-Energy and Long-Life Aluminum-Sulfur Batteries. *Angew Chem Int Ed* **61**, e202202696 (2022).

67. Zhao Y*, et al.* Initiating a Reversible Aqueous Zn/Sulfur Battery through a "Liquid Film". *Adv Mater* **32**, 2003070 (2020).

68. Zhang H*, et al.* Redox Catalysis Promoted Activation of Sulfur Redox Chemistry for Energy-Dense Flexible Solid-State Zn-S Battery. *ACS Nano*. **16**, 7374-7351 (2020).
